# Experiences of Emergency Nurses Providing End‐of‐Life Care During the COVID‐19 Pandemic

**DOI:** 10.1111/jan.16749

**Published:** 2025-01-16

**Authors:** Alysha Cowley, Julia Morphet, Kimberley Crawford

**Affiliations:** ^1^ School of Nursing and Midwifery, Sub‐Faculty of Health Sciences, Faculty of Medicine, Nursing and Health Sciences Monash University Clayton Victoria Australia

**Keywords:** COVID‐19, Emergency department, Emergency nursing, End‐of‐life care

## Abstract

**Aim:**

To explore the experiences of emergency nurses providing end‐of‐life care during the COVID‐19 pandemic.

**Design:**

A qualitative descriptive study.

**Methods:**

Data were collected between May and August 2023. Individual, semi‐structured interviews were conducted face to face or by videoconferencing with a purposive and snowballed sample of 11 emergency nurses. Data were analysed using thematic analysis.

**Results:**

There were four main themes identified: (1) Isolation and loneliness that emergency nurses felt when providing end‐of‐life care throughout COVID‐19. (2) Comparison of Care Relating to Communication; whereby emergency nurses compared the provision of end‐of‐life care before COVID‐19 to end‐of‐life care provided during the pandemic. (3) Frustration and guilt: emergency nurses felt when providing end‐of‐life care. (4) Teamwork: participants interviewed explored the meaning of having a supportive team when providing end‐of‐life care.

**Conclusions:**

This study explores in‐depth experiences of emergency nurses providing care to those who were dying during the COVID‐19 pandemic. This study identified that emergency nurses were impacted emotionally by providing end‐of‐life care during the pandemic, and ongoing support is likely to be needed. This study also reinforced the strength of teamwork amongst emergency nurses.

**Patient or Public Contribution:**

No patient or public contribution.

**Summary**

- What does this paper contribute to the wider global community?
  ○ Understanding that emergency nurses require ongoing emotional support due to the impacts that the provision of end‐of‐life care during the pandemic had.
  ○ Need for a robust communication system for family and/or carers to remain informed of end‐of‐life care planning.
  ○ The importance and need for family and/or carer presence for those being provided end‐of‐life care in the emergency department.

## 1 Introduction

The emergency department (ED) is an essential component of the healthcare system. Acting as the entry point to the hospital, EDs care for patients at all stages of their life, from birth until death. The ED has long been a place where end‐of‐life care is provided, with emergency nurses playing a key role in providing this care. In Australia, approximately 51% of patients die in a hospital environment (Australian Bureau of Statistics 2021), therefore necessitating the need for nurses to provide person‐centred end‐of‐life care. As demand on EDs to provide end‐of‐life care increases Australia‐wide (Tiah et al. 2023), the demands of emergency nurses to provide compassionate end‐of‐life care also increase. It has been well evidenced that there are challenges in providing end‐of‐life care to patients in the ED (Alqahtani and Mitchell 2019; Robson and Craswell 2022), such as communication, the ED environment, and the emotional anguish of nurses providing end‐of‐life care (Decker, Lee, and Morphet 2015; Hogan et al. 2016). The COVID‐19 pandemic exacerbated many of these challenges but also brought forth its own set of challenges for emergency nurses providing end‐of‐life care.

In Victoria, Australia, tight restrictions were implemented that affected people's ability to travel, socialise, and visit family in the hospital environment. In Australia, between 2020 and 2022, the state of Victoria saw the largest number of deaths due to COVID‐19 at 4487 deaths (Australian Bureau of Statistics 2023). This number impacted both hospitals and EDs, as well as emergency nurses taking care of patients that were dying during this time.

## 2 Background

End‐of‐life care, as defined by the Australian Commission on Safety and Quality in Healthcare (ACSQH), “includes physical, spiritual, and psychosocial assessment, care, and treatment delivered by health professionals and ancillary staff” (Australian Institute of Health and Welfare 2016). Patients are considered to be approaching the end of their life when it is likely they will die within the next 12 months, and it often includes those where death is imminent (Australian Institute of Health and Welfare 2016). ED is frequently the place where end‐of‐life care is initiated or where patients who are experiencing discomfort in the face of a life‐limiting illness present for care.

Previous studies focused on how the ED impacted end‐of‐life care and nurses who were caring for those that were dying. These studies mainly focused on the environment of the emergency department (Decker, Lee, and Morphet 2015; Díaz‐Cortés et al. 2018; Kongsuwan et al. 2016), the ability to identify the needs of the patients who were dying (Decker, Lee, and Morphet 2015; Omoya, De, and Breaden 2022; Wolf et al. 2015), the educational needs of emergency nurses (Díaz‐Cortés et al. 2018; Tse, Hung, and Pang 2016; Wolf et al. 2015), the emotional impact that caring for dying patients had on emergency nurses (Decker, Lee, and Morphet 2015; Granero‐Molina et al. 2016; Hogan et al. 2016), and the challenges of communication in the emergency department (Díaz‐Cortés et al. 2018; Granero‐Molina et al. 2016; Omoya, De, and Breaden 2022).

Previously, the ED was seen as an environment that was not conducive to a peaceful death and posed a challenging setting to provide dying patients with comfort and privacy (Díaz‐Cortés et al. 2018; Kongsuwan et al. 2016; Tse, Hung, and Pang 2016). Private rooms, separated from the noisiness of ED, were seen as beneficial to allow loved ones space for a peaceful death (Díaz‐Cortés et al. 2018; Kongsuwan et al. 2016; Tse, Hung, and Pang 2016). Once a patient was identified to be dying, their care was often expedited to a ward‐based environment, where they could be provided with a single room that facilitated privacy and peace for end‐of‐life care to be delivered, which was more likely to align with the wishes of the patient (Decker, Lee, and Morphet 2015).

Research has shown that emergency nurses often feel they lack the means and the time to provide compassionate care to those that are dying (Hogan et al. 2016). A lack of time and resources, as well as the inability to prioritise care due to the demands of the ED, posed significant challenges to end‐of‐life care in the ED (Decker, Lee, and Morphet 2015; Hogan et al. 2016; Tse, Hung, and Pang 2016). Research has also considered how challenges exist in the ED for identifying the needs of the dying. Discussion centred on emergency nurses' feelings towards the difficulty in symptom management due to the hesitancy of medical staff to prescribe medications (Kongsuwan et al. 2016; Tse, Hung, and Pang 2016), leading to delays in pain relief and symptom control. A lack of knowledge of patients' wishes or advanced care directives had previously impacted emergency nurses' ability to identify the needs of dying patients (Wolf et al. 2015). Due to perceived lack of knowledge and skill, emergency nurses felt they struggled to provide holistic and comprehensive end‐of‐life care that addressed patients' needs (Díaz‐Cortés et al. 2018; Wolf et al. 2015). Nurses reported that their care centred on task completion, rather than person‐centred end‐of‐life care.

End‐of‐life care has a large emotional toll on nurses due to the distress felt by the family and/or carers, as well as the patient (Granero‐Molina et al. 2016; Hogan et al. 2016). Emergency nurses identified feelings of distress when they felt they had not provided patients with a ‘good death’ (Decker, Lee, and Morphet 2015). Although providing end‐of‐life care could be distressing, emergency nurses also felt that this care could have a positive impact on the patient and their family, whereby they were able to offer support, in an otherwise constantly changing environment (Díaz‐Cortés et al. 2018; Hogan et al. 2016; Tse, Hung, and Pang 2016).

Research conducted prior to COVID‐19 focused on end‐of‐life care in the ED and how emergency nurses experience providing care to dying patients. There has, however, been little research into how end‐of‐life care changed for emergency nurses throughout COVID‐19 and how the pandemic affected the provision of end‐of‐life care. For the ED, COVID‐19 had significant implications, especially for end‐of‐life care. EDs were challenged with the demands of the pandemic, and from the perspective of emergency nurses, changes were occurring frequently (Keyes et al. 2021). Due to the demands and lack of accessibility to primary health care, the ED was often responsible for end‐of‐life care during the pandemic (Sena and De Luca 2022). It is important to consider the perspective of emergency nurses in providing end‐of‐life care during COVID‐19 to better understand their experiences as well as how best to support the emergency nursing workforce providing end‐of‐life care and to improve end‐of‐life care in the future.

The aim of this study was therefore to explore the experiences of emergency nurses providing end‐of‐life care during the COVID‐19 pandemic.

## 3 Methods

### 3.1 Research Design

This research study employed a qualitative descriptive approach, aiming to gain a better understanding of how emergency nurses experienced providing end‐of‐life care during the COVID‐19 pandemic. A qualitative descriptive approach was chosen for this study due to the rich and in‐depth data that was needed to address the aims of this study. Utilising this research design recognised that each emergency nurse's experience with end‐of‐life care was subjective, and descriptions about their own perceptions and understanding of the provision of end‐of‐life care during COVID‐19 were needed (Sandelowski and Barroso 2003). The COREQ 32‐item checklist was utilised to guide the development of the manuscript (Tong, Sainsbury, and Craig 2007).

### 3.2 Study Setting and Recruitment

Participants were recruited from Victoria, Australia. Nurses who were working in the ED between 2019 and 2022, had worked as emergency nurses for more than 2 years, and who had provided end‐of‐life care during the pandemic were eligible to participate in the study. The reasoning for only including Victorian emergency nurses was that Victoria experienced the largest and longest lockdown periods throughout the COVID‐19 pandemic globally (Griffiths et al. 2022), which impacted both EDs and the emergency nurses caring for patients requiring end‐of‐life care.

Participants were recruited through both purposive and snowball sampling. Researchers sought assistance from the College of Emergency Nursing Australasia (CENA) to assist with recruitment. CENA distributed emails to their Victorian members, notifying them of the study. A flyer was also distributed to those enrolled in a postgraduate emergency nursing program at one of Australia's largest nursing schools. Recruitment documents included a QR code or link to a Qualtrics form; participants wishing to be included in the study could provide their email address, which allowed the researcher to be in contact. Participants were provided with an explanatory statement about the study via the Qualtrics form. A total of 11 participants responded to the recruitment process and met the inclusion criteria, so were therefore included in the study.

### 3.3 Data Collection

Data were collected between May and August 2023. The interviewer contacted the participants, who had expressed an interest via the Qualtrics form, to arrange a convenient time and day to be interviewed. The authors created an interview guide informed by the literature, with semi‐structured interview questions (Table 1). The interviews were conducted by the primary author either face to face or via videoconferencing using the digital Zoom platform. This was chosen, based on convenience, by the interviewee. All interviews were voice recorded.

**TABLE 1:** Interview questions guide.

1.	Can you share with me an experience you had caring for a dying patient during COVID‐19?
	If yes and shared, why did you choose this particular experience to share?
2.	Can you tell me what it was like to care for someone who died in the emergency department during COVID‐19?
3.	How did COVID‐19 impact the provision of end‐of‐life care?
4.	What were the factors that facilitated caring for someone who was dying in the ED during COVID‐19?
5.	What were the factors that hindered caring for someone who was dying in the ED during COVID‐19?
6.	What changes did you experience when communicating with patients during COVID‐19 in ED?
	a. What were the challenges to this communication?
	b. What were enablers to this communication?
7.	What changes did you experience when communicating with family members during COVID‐19 in the ED?
8.	What support were you provided after caring for a dying patient?
9.	What support would you have liked to have been provided?
10.	In your utopian ED, where funds and resources are infinite, how would you like care of the dying in the ED to be provided?

The interviews lasted between 28 min and 1 h. The first author (AC) conducted all of the interviews, with the last author attending the first two interviews (KC). The last author assisted the first author, as a junior researcher, to assist in providing guidance and feedback. The lead author asked all of the interview questions, with the last author asking follow‐up questions at the end of the interview. Permission was sought from the participants to have two researchers present for these interviews.

Two of the authors had been emergency nurses for over a decade, and therefore throughout the analysis process, it was important to identify any biases or views that may have influenced the interpretation of the findings (Darawsheh 2014). Both researchers routinely reflected on any assumptions they may have had or expectations of information that may not have derived from the interviews. This was important to ensure trustworthiness in the generation of themes and thematic analysis. As data analysis and collection occurred concurrently, data collection ceased when ‘information power’ was reached (Braun and Clarke 2022). It was decided during the iterative data collection and analysis phase that information power, in this context, had been reached due to the richness of the responses and data provided by the participants that was able to address the aim of this study.

### 3.4 Data Analysis

The data collected from the semi‐structured interviews were transcribed verbatim using Microsoft 360 transcribing technology and were checked for accuracy. Braun and Clarke's six steps of reflexive thematic analysis were utilised to generate codes and themes discussed in the results section of this paper (Braun and Clarke 2022). Following this six‐step process, the researchers familiarised themselves with the data. The three authors coded the transcribed data individually, with one researcher coding all of the interviews and two researchers coding half of the interviews each using NVivo and an iterative process to generate codes. The codes were then shared amongst the three authors, themes were generated, developed, and reviewed. After being reviewed, the themes were named and refined, and finally the write‐up process was completed. The themes were derived from the data and were not identified prior to analysis and were agreed on by all three researchers through discussion of the importance of the themes.

### 3.5 Ethical Considerations

Ethics approval was sought through Monash University Human Research Ethics Committee, Victoria, Australia (Project Number: 36816).

Participants were provided with an explanatory statement prior to the interviews being conducted to inform the consent process for the participants. Participants gave verbal consent to be involved in the study. Due to the discussion of death and dying, participants were guided to where they could access support, should they find the interviews distressing, and were informed that interviews could be ceased at any time if they felt overwhelmed. To protect the anonymity of the participants, each participant was de‐identified by being assigned a number.

### 3.6 Trustworthiness

In order to ensure trustworthiness within the study, the researchers followed a logical research process that was well documented (Nowell et al. 2017). The researchers also implemented Lincoln and Guba's (1982) four criteria for trustworthiness: credibility, transferability, confirmability, and dependability (Lincoln and Guba 1982). To address dependability and show coherence between the methods and the findings, the interviews with the participants were audio recorded and transcribed verbatim. The researchers used triangulation and ensured that the conclusions were derived from the data by collectively being involved in the coding and theming process of thematic analysis. Participant quotes were used in each theme to show evidence that the themes were generated from the participants own words. To further address trustworthiness in this research, contextual information relating to the methods and participants is provided.

## 4 Findings

### 4.1 Participant Demographics

A total of 11 emergency nurses were interviewed. All participants identified as female. The median age of the nurses was 30 years old, with ages ranging from 24 to 42 years old. The median time working as an emergency nurse was 5 years, with the time working as an emergency nurse ranging from 2 to 20 years. All participants held a bachelor's degree in nursing. Most of the participants also held a higher qualification; two held a postgraduate certificate, five a master's degree and two an honours degree. Participants worked across a range of public hospital emergency departments, most of which were located in metropolitan Melbourne (*n* = 8), with three participants working in regional emergency departments.

Following the data analysis, four themes were developed. These themes were isolation and loneliness, comparison of care relating to communication, frustration and guilt, and teamwork.

#### 4.1.1 Theme 1: Isolation and Loneliness

The first theme, of isolation and loneliness, highlighted the emergency nurses' feelings of segregation. Participants openly discussed the multifaceted way that the COVID‐19 pandemic isolated and segregated patients from their loved ones, isolated themselves from their colleagues, and isolated them, as emergency nurses, from their patients. It was also evident from the participants' discussions that there was a physical barrier when providing end‐of‐life care that also caused feelings of isolation and loneliness. These physical barriers included isolation cubicles where participants were caring for dying patients behind a closed door, personal protective equipment (PPE) that formed a barrier to providing compassionate care, and colleagues not physically being able to share meal breaks or debrief with each other following a shift or difficult presentation. As the following participant alludes to, they felt empathy for patients that were isolated by being in an isolation cubicle and were not surrounded by loved ones.

It was very different. Very new. You kind of felt a bit isolated… I think for the patients a bit more isolated as well. Because they're in this room with the closed door… and sometimes they're just in there with no family members at all, because we didn't want family members in COVID rooms… It's probably very isolating and lonely; even as nursing staff, you get stuck in a room. You often don't get people coming in and checking on you probably as often as we'd like. (P3)

Interviews revealed that prior to the pandemic, participants often felt connected to dying patients and their loved ones. COVID‐19 caused a barrier in the ability for emergency nurses to build connections and establish a caring relationship, whereby they felt disconnected from patients and their families. Emergency nurses empathised with how patients and families experienced the pandemic, and how they too must have felt segregated from their own loved ones. Participants discussed how prior to COVID‐19, the number of visitors for patients nearing the end of their life was often unlimited, and loved ones were encouraged to attend the ED to offer comfort and support during end‐of‐life care. It was evident in the interviews that participants were distressed by the isolating nature of COVID‐19 when providing end‐of‐life care. Many participants became teary and upset during the interviews, revealing how when they reflected on their experiences of providing end‐of‐life care during the pandemic they were still impacted by the provision of that care. As the following participant describes, the feelings of isolation and segregation they were experiencing often stemmed from feeling disconnected from both the patients and their families during the pandemic. “We felt, you know, we couldn't. We couldn't let family in. Oh God, like we couldn't let family in, and to have somebody die or be dying alone without that support was just horrendous.” (P11).

Due to the nature of the presentations of dying patients, many were considered to have respiratory symptoms and therefore isolated from others in the ED. As a result, the emergency nurses who cared for these patients were also isolated from all other people in the ED, and they described the struggle of isolation on consecutive shifts and felt exhausted by the thought of being alone for hours at a time, day after day. One participant described the feelings of being ‘stuck’ in isolation rooms, not seeing their colleagues for hours, and the impact this had on future shifts.

You're stuck in an ISO (isolation) room for six hours and just understanding the way that oppression of being stuck. And the impact that has when you don't get to see someone, another person, and you don't get assistance from your team, that literally someone checking in on you can make or break a shift for you, and then that makes or breaks how you go home and you recover from that shift, which absolutely changes who you are when you come into the next shift. (P7)

Aside from segregation from their colleagues, participants spoke of how loneliness and isolation impacted the care that was provided to patients at the end of their life. As discussed by the participants, this was due to restrictions on visitors or difficulties with staffing that led to care, such as moving the patient in a bed or cleaning a patient, being left to one nurse, and the inability to show compassion due to PPE hindering physical touch, as the following participant identifies: “There were so many things that changed that communication, just wearing masks… just decreases half your facial expressions and emotions that you can express. Physical touch” (P1).

It was evident from the interviews conducted that emergency nurses harboured a sense of guilt from refusing entry to the ED to family members and friends when they were visiting patients who were dying. Tight visitor restrictions that were implemented in Victoria prevented a large number of family members from being in attendance when a loved one was dying, further exacerbating how isolated patients and family would have felt. One participant spoke of the uncomfortable discussion of turning loved ones away when visitor restrictions inhibited the ability of patients to be accompanied by family or friends.

And I felt like, you know, you feel like your gut gets pulled out of you a little bit when you have to say to a family member, You can't be in the room with them; it wasn't in that patient's best interest. (P7)

The restrictions on visitors meant that emergency nurses were often the only people providing company and a hand to hold when these patients were facing death. The following two participant quotes reveal how the emergency nurses considered their own feelings of loneliness, but also those feelings of the patients: “It was just really lonely. Yeah. I think for the patient. And for the staff as well.” (P5) Another participant said, “(I was caring for) an intubated patient, and as the rules didn't allow in Victoria for family members to visit that particular person, I was the only one that was allowed in the room” (P8).

#### 4.1.2 Theme 2: Comparison of Care Relating to Communication

The second theme developed from the data was the comparison of end‐of‐life care during COVID‐19 with that of care prior to the pandemic. Comparison of care for emergency nurses was how they saw end‐of‐life care change or mould to the demands placed on the ED during the pandemic.

The participants discussed changes to communication with patients, loved ones, and their colleagues in the ED during COVID‐19, as well as the implementation of new technologies to assist with communication. Communication was one of the areas of end‐of‐life care where emergency nurses saw significant changes. Due to the inability of family members and loved ones to attend the ED, or when tight restrictions were implemented, participants shared that much of the communication with family was completed over the phone. “There was a lot more phone communication. I would say. Because you'd have multiple phone calls coming in from… different people” (P5).

Participants' views on phone communication differed. Some emergency nurses discussed that phone communication seemed to take away from the time they were spending with patients, whilst others found it a valuable way of updating family members and loved ones with information pertaining to the patient's condition. Participants also found that they were needing to update multiple family members, rather than just facilitating communication with one family member, further contributing to time that could have instead focused on providing end‐of‐life care. As the following participant discusses, time was often spent communicating with family members over the phone: “But we definitely spent more time on the phone with them. Obviously if a family couldn't be present, and then the patient was asking, Oh, can you give my wife an update?” (P1).

One of the other challenges of phone communication, identified by the participants, was maintaining privacy and confidentiality. In the context of end‐of‐life care, some patients were not able to give consent to allow for updates on their condition to be conveyed to family members. This concerned many of the emergency nurses and brought into question whether they were breaching the privacy of that patient, should they not wish for information to be shared, as the following participant discusses:

I found it really tricky trying to keep the family updated and knowing the family. Like, with the confidentiality stuff and all that stuff, who I can say what to. What family members are going to react to what I say? … I found it really difficult to keep the family updated. (P9)

There was also often confusion about what aspects of the patient's condition could be shared, especially in the case of end‐of‐life care. Many of the participants were unsure whether it was appropriate to tell a loved one that the patient they were caring for was dying. As the following participant discusses, phone communication did bring into question whether pertinent information could be shared: “Then again, if you've got an end‐of‐life patient who can't tell me that it's OK for us to give details, and sometimes your next of kin details aren't up to date. So that gets complicated” (P6). Similarly, participant nine spoke of confusion relating to which aspects of the patients details and care could be communicated over the phone. ‘You can tell this person because they're the next of kin, but then you got other people ringing up and like, oh, I'm the daughter. I'm the son, and you're like, Well, can I tell you, can't I tell you? Yeah, I found that hard with the patient.’ (P9).

Participants spoke of technologies that were introduced throughout COVID‐19 to assist with communication. Interviews revealed that many of these technologies were to aid communication between nurses caring for patients in an isolation cubicle with those outside. These technology strategies attempted to mitigate the need for PPE wastage, as nurses who were not directly caring for the patient could avoid entering the room and therefore did not need to don PPE. Many participants that were interviewed spoke of an intercom‐style tool being installed in their EDs to enable requests for assistance, equipment, or required resources. Participant three explains the concept in their ED: “A lot of the time we had the speakers in and out of the room, so you're communicating with other nursing staff on the speaker” (P3).

Similar to phone communication, comments and discussions about the two‐way speaker technology were met with mixed reviews. Some emergency nurses thought this aided communication, whilst others commented on how often this technology was faulty or would only last for a limited time before it was replaced or no longer in use. As the following participant states, the use of the two‐way speaker at times delayed care for the patients;

I'm just remembering back to our anterooms. A lot of the time we had the speakers in and out of the room, so you're communicating with other nursing staff on the speaker; they didn't actually come into the room, so sometimes there was a lot of delay in getting even things like a blanket or something for a patient because, you know, it's time. (P3)

Alternatively, the following participant considers how changes to technology hindered care, and rather than the helpfulness of the technology, as it was intended, the call system and phone communication were detrimental in providing end‐of‐life care, “The phone stopped working, and no one's answering the doorbell. Like, I literally can't do anything else” (P7).

#### 4.1.3 Theme 3: Frustration and Guilt

The third theme identified from the interviews was the theme of frustration and guilt. Frustration, in the context of emergency nurses providing end‐of‐life care during the COVID‐19 pandemic, was seen as a feeling of anger and sometimes a feeling of defeat when trying to provide effective and compassionate end‐of‐life care. The frustration that was revealed by the emergency nurses was not directed at providing the end‐of‐life care itself but rather at the barriers and factors that hindered this care during the pandemic. Participants also felt guilt when caring for end‐of‐life patients through the pandemic. Their guilt was multifaceted and related to early initiation of palliation rather than life‐sustaining treatment, time constraints when trying to provide end‐of‐life care, and staffing resources that were challenging throughout the pandemic.

The feeling of frustration, as discussed by the participants, was linked, largely, to unpredictable and frequent changes to policy. Participants frequently discussed how changes to policies and procedures that related to visitor restrictions, PPE, and care provision impeded their ability to provide holistic end‐of‐life care. Participants described that the frequency of policy changes, and subsequent time taken to keep up to date with changes, impacted the time available to provide patient care, which was a source of frustration. As the following participant reflects, there was a level of frustration at how care was lacking for patients and how policies and procedures hindered end‐of‐life care: “I think the thing that I found most difficult was the fact that everything was changing all the time and so I didn't feel proficient in my practice because I was like, What's going on? Like everything is changing.” (P4).

In reference to participants considering and discussing the frustrating and ever‐changing climate of policy and procedure changes, participants also identified not only a sense of frustration but a sense of guilt for caring for dying patients during COVID‐19. The inability that emergency nurses felt, coupled with a sense of helplessness, stemmed from the initiation of palliation of patients, rather than life‐sustaining treatments. This was portrayed by the participants interviewed to contribute to poorer patient outcomes, leaving emergency nurses not only frustrated but still harbouring anger for the palliative trajectory of many patients. Some of the outcomes that were discussed by the participants included unexpected or hastened death, delayed time for administration of comfort medication or review by medical staff, and delayed transfer to the ward. The following quote, by participant nine, is indicative of this feeling of helplessness, as well as the frustration at providing inadequate care. This quote was in context to witnessing a patient die due to not implementing aerosol‐generating procedures and non‐invasive ventilation, attributed to the policies surrounding this during the pandemic.

I was looking after a gentleman that came in with pneumonia. He didn't have a heap of comorbidities. He was a bit overweight, and unfortunately, he was made to have end‐of‐life care because we couldn't do BIPAP (Bilevel Positive Airway Pressure, commonly used to assist with spontaneous respiration) because we weren't allowed to do aerosol‐generating procedures. So it was quite hard to watch somebody die when we could actually do something, but we weren't allowed. (P9)

The frequent policy changes, as mentioned previously, also related to changes that often occurred to rules regarding PPE use. Participants outlined the toll that PPE had on providing end‐of‐life care throughout the pandemic and the frustration this caused. PPE became a time‐consuming necessity in the care of dying patients due to the patients often presenting with respiratory symptoms. The donning and doffing process took significant and precious time away from the nurse's ability to provide end‐of‐life care that was both holistic and compassionate. PPE also caused hesitation for the nurses entering the cubicle of patients at the end of their life. This hesitation was discussed by the participants, who contributed to substandard care for dying patients. As the following participant explains, PPE caused challenges for emergency nurses providing end‐of‐life care, causing significant delays to care.

It was definitely challenging from the sense of having to gown up and do PPE and get in there. Sometimes I felt like we delayed treatment because we didn't want to or were trying to cluster their care. (P9)

Participants empathetically spoke of the inadequacies of end‐of‐life care that were not present prior to the COVID‐19 pandemic. Many participants spoke of a sense of guilt in the care they were providing to the dying, and many attributed this to time constraints and staffing. A lack of time was a key area where participants compared care during COVID‐19 to prior, as the emergency nurses interviewed identified that quality and compassionate end‐of‐life care took significant time, which was not available during the pandemic. An inability to find other staff or colleagues, as experienced by the participants interviewed, showed that others also lacked time to assist with basic nursing care that required multiple staff, such as attending to pressure area care or changing a soiled patient, imperative to care of the dying. As the following participant discusses, they felt unable to provide optimal care to the patients due to a lack of time and resources.

I think it's more just frustration on behalf of the patient that they haven't received the care that they should have, and I think it was more frustration at the system that we are so overwhelmed and overrun that we don't have time to be able to provide this care. (P2)

As one participant also explains:

There were other aspects that were quite frustrating, like not having adequate staffing, that you couldn't find someone to help you roll your patient, or to get like, a double check to medication out of the cupboard with you for them. (P1)

#### 4.1.4 Theme 4: Teamwork

The final theme that emerged from the participants' interviews was that of a strong sense of teamwork and camaraderie that participants felt when caring for end‐of‐life patients during the COVID‐19 pandemic.

Historically, emergency nurses have worked in teams in order to deliver a high standard of care to those that present to the ED, not just to those that are dying (Han and Roh 2020). Teamwork is not something that is new to emergency nursing in the wake of the pandemic; however, teamwork is what strengthened the ability of the participants to provide difficult and complex end‐of‐life care in an environment that was less than ideal to provide that care. The theme of teamwork brought forward a positive aspect and discussion from the participants, in a time where emergency nurses found providing person‐centred end‐of‐life care difficult. Many participants considered how they drew strength from their team when dealing with an unexpected death or were struggling with caring for patients at the end‐of‐life. The following quote from participant six outlines the support that they received from the other emergency nurses in their team when support was hard to come by: “We emphasised that team aspect. We made sure that we were looking after each other and checking in with each other.” (P6).

Despite challenges in providing care to dying patients throughout COVID‐19, participants found solace in the knowledge that although there was a sense of loneliness when caring for those that were dying, they had support. This support was offered by fellow emergency nurses, as well as the team of staff working in the ED. As the following participant discusses, there was a strong focus on colleagues looking out for and caring for each other throughout the pandemic.

I think having that collective experience allowed us to have a better sense of teamwork because we were very much focused on caring for one another, checking in with one another. I think that was a really good environment to work in. (P2)

Participants acknowledged the difficulties of providing end‐of‐life care during the pandemic; however, they were able to draw on their own experiences and credited their colleagues for being a strong support system. Many participants identified the comfort they felt in the knowledge they had a team they could lean on, but also considered how they were, alternatively, the person other nurses could lean on. There was a reciprocal relationship amongst the participants and their team members, whereby on the days they were struggling, their team carried them, and on days their team members were struggling, they carried their team. The following participants' quote depicts this relationship: “I think I felt safe. I had a good team on, and she said, ‘Absolutely, I've got your back’, she said, ‘I'll probably need the same tomorrow’.” (P7) and “I've got a good team. We've got a good team.” (P6).

## 5 Discussion

This research identified four themes that affected the provision of end‐of‐life care during the COVID‐19 pandemic. Participants discussed how physical isolation impacted their ability to be supported by their colleagues and resulted in loneliness. This was further exacerbated as the participants were often the only people present to hold patients' hands in the face of death. It was evident from the interviews conducted that participants compared the care they provided prior to the pandemic to the care they provided during the pandemic, identifying phone communication and technology changes as factors that were implemented to respond to the needs of the pandemic. As well as feelings of isolation and loneliness, participants experienced a sense of frustration in relation to the provision of end‐of‐life care during COVID‐19. This frustration was due to the numerous changes to policies and procedures, the time‐consuming nature of PPE that detracted from time they spent providing end‐of‐life care, and the necessity to turn away loved ones that wished to visit a family member. Although COVID‐19 presented many challenges for emergency nurses when providing end‐of‐life care, participants also acknowledged the positivity of the team environment that they worked in, and the trust they had in their colleagues. Participants attributed their ability to remain resilient and provide person‐centred end‐of‐life care to the team that surrounded them, finding comfort in the knowledge that on tough days, their fellow emergency nurses and colleagues would respond by providing support and guidance.

Much of the research, conducted prior to COVID‐19, focused heavily on the ED itself as being an unfavourable environment to provide end‐of‐life care (Decker, Lee, and Morphet 2015; Kongsuwan et al. 2016; Wolf et al. 2015). Research identified that the dynamic, and often loud setting was not cohesive with a peaceful and dignified death (Díaz‐Cortés et al. 2018; Kongsuwan et al. 2016; Tse, Hung, and Pang 2016). The unpredictability of the ED, coupled with providing privacy for those that are dying, made the ED a difficult setting for the provision of end‐of‐life care (Wolf et al. 2015). Interestingly, the participants interviewed in this research did not discuss in depth the challenges of the ED environment when providing end‐of‐life care during the pandemic. Aside from discussing the isolation of single rooms for patients with symptoms of COVID‐19, there was little indication from the interviews conducted that the environment was a factor when considering how end‐of‐life care changed during COVID‐19. The omission of this information is a stark contrast to the emphasis that had previously been placed on the ED environment and the way healthcare providers viewed the ED as a less‐than‐ideal place for the provision of end‐of‐life care. There was no evident reason for the lack of participant focus on the environment of the ED as a challenge to providing end‐of‐life care; however, due to the competing priorities of the pandemic, this may not have been seen as the strongest contributing factor to end‐of‐life care.

Communication is a key factor when providing end‐of‐life care in the ED. Factors that impacted communication prior to the pandemic, in relation to end‐of‐life care, were discussed in relation to loved ones not understanding a patient's wishes, when they were brought to the ED, or there was miscommunication amongst members of the multidisciplinary team (Granero‐Molina et al. 2016). This study highlighted a communication challenge that was difficult to mitigate. Instead of a lack of understanding of the patient's wishes, or miscommunication occurring, much of the communication regarding a dying patient needed to be relayed over the phone. Participants saw this form of communication as taxing on their time and ability to provide end‐of‐life care. COVID‐19 brought new and unforeseen limitations to visitation, whereby phone communication was a necessity to keep loved ones involved in decision‐making and care. Aside from phone communication with loved ones, communication amongst staff also saw many changes, including the implementation of technology that allowed colleagues to communicate with each other when the provision of care took place in an isolation room. These changes to technology were not just siloed to the ED during the pandemic but were also prevalent in other health care environments, such as the use of telehealth in primary health care (Doraiswamy et al. 2020) and the implementation of virtual EDs for the ambulance service (Sri‐Ganeshan et al. 2023). Participants saw technology changes as both a hindrance and a help. Often the technology was not functional, or it was difficult to catch the attention of others to implement the use of this communication system. Overall, COVID‐19 saw a shift from the difficulties of understanding what it was the patients wanted at the end of their life to communication that occurred via phone and at a distance. Moving forward, a more robust communication system, or technology implementation, may be needed for emergency nurses to communicate with loved ones. If a pandemic were to occur again, or simply for patients that require isolation, implementing a functioning system for communication is needed.

It was evident throughout the interviews that participants empathised and carried a large emotional burden, often being the sole providers of comfort and companionship for patients at the end of their lives and their families. Participants repeatedly discussed empathy and putting themselves in the shoes of loved ones who were unable to be present. Although research suggests that emergency nurses often possess personality traits, such as grit, self‐leadership, and strong communication skills (Jeong and Lee 2023), meaning they may be viewed as unbreakable and unaffected by providing end‐of‐life care. It was clear that the participants were impacted by death during COVID‐19, regardless of these personality traits. In light of these feelings, even long after care was provided to patients that evoked these emotions, it may be beneficial for emergency nurses to be supported in a more meaningful way. This support may include simple strategies, such as debriefing post a patient's death or allowing staff to take breaks together to address the feelings of loneliness that emergency nurses experienced and support the workforce as a whole.

Many patients died alone throughout the COVID‐19 pandemic, and it was obvious that emergency nurses continued to be impacted by this knowledge. The loneliness and isolation that the participants alluded to were also acknowledged as feelings that the patients must have felt. In line with previous research, participants felt an emotional toll and grief when providing end‐of‐life care during the pandemic (Granero‐Molina et al. 2016; Hogan et al. 2016). Although these feelings of grief were a common thread both before and during the pandemic, it was the difficulty of witnessing a patient die alone that appeared to trouble the participants most. Unlike prior to the pandemic, when visitor restrictions were not enforced, nurses were the people holding the hands of those that were dying during COVID‐19. It was distressing to a number of participants that they, only having known the patient for a brief window of time, were the sole source of companionship in the face of death.

Finally, emergency nurses have long valued working in a team environment, possessing personality traits of openness to experiences and agreeableness, meaning they are considerate and conscientious of others' experiences (Kennedy, Curtis, and Waters 2014). Historically, emergency nurses value supporting one another and sharing experiences, whilst also understanding the importance of working and learning together (Suryanto, Plummer, and Copnell 2016). It is this teamwork that many participants saw as invaluable throughout the pandemic when providing end‐of‐life care. COVID‐19 presented feelings of segregation, isolation, and loneliness, so it may appear contradictory that emergency nurses were able to identify teamwork as a positive aspect of providing end‐of‐life care. Colleagues were prevented from debriefing following difficult patient encounters, including those that were dying, due to density limits and room restrictions. Despite this, it was encouraging that many participants were able to identify and discuss the way the emergency nursing team, albeit strong before COVID‐19, was a pillar of strength for aiding colleagues and staff to provide the best care they could to dying patients. This demonstrated the strength of the emergency nurses as a collective team and highlights previous research identifying that emergency nurses feel a greater sense of job satisfaction, as well as a reduction in stress, when teamwork is present in the ED (Grover, Porter, and Morphet 2017).

Despite the COVID‐19 pandemic being currently under control, it is important to acknowledge the transferability of the findings of this study. For emergency nurses that provide end‐of‐life care, ongoing support to mitigate emotional distress is needed to enable them to continue to provide high‐quality, patient‐centred care of the dying.

Should another pandemic present, whereby patients are needed to be isolated from one another, and family members are not allowed to visit loved ones, the ability to be flexible in the face of death is required. Participants reiterated their feelings of guilt about turning family members away, and it is important that patients at the end of their life are able to be surrounded by loved ones.

### 5.1 Strengths and Limitations

Participants in this study were honest and open in sharing their experiences of providing end‐of‐life care throughout the pandemic. They were able to express their own feelings about the changes and implications of end‐of‐life care provision throughout the pandemic. There were some recognised limitations from the study. Data were collected in 2023, and the experiences that nurses had during COVID‐19 related to the years 2020–2022. Nurses were asked to reflect on these experiences up to 3 years after the fact. Most participants did, however, have vivid recollections of providing end‐of‐life care during this time, evidently representing the long‐lasting influence these experiences had.

### 5.2 Implications for Practice and Future Research

It was evident from the interviews that many emergency nurses continue to be impacted by their provision of end‐of‐life care at this time. The emotional impact and challenging nature of implementing visitor restrictions, as well as policies and procedures that affected the way care was provided, have stayed with many of the nurses who provided end‐of‐life care. It is important to consider, for the future, how emergency nurses can be supported on a longer‐term basis to address the toll that this continues to have. Psychological stress and emotional impacts need to be addressed to provide a protective mechanism for nurses after the pandemic (Ghorbanzadeh et al. 2022). The importance of supporting the nursing workforce, now more than ever, may address workforce planning and attrition. Further research into emergency nurses' experiences of COVID‐19 is needed to further explore the support that may be necessary in the future.

Many participants discussed the importance of the presence of loved ones in the provision of end‐of‐life care. This was seen as a non‐negotiable for ensuring the patients were not isolated or alone. As well as the breakdown in communication that was evident amongst staff and family during the pandemic, it is important that careful consideration is given to how loved ones are relayed information and are allowed to visit patients who are dying, should another pandemic present. To address feelings of isolation and loneliness, further research into a robust system for communication is needed that allows family members to remain connected to their loved ones.

## 6 Conclusion

Prior to this study being conducted, little was known about emergency nurses' experiences providing end‐of‐life care during the COVID‐19 pandemic.

This study reinforced the knowledge and previous feelings of emergency nurses valuing working in a team and how they saw their colleagues as a strong support system for the emotional toll of providing end‐of‐life care during the COVID‐19 pandemic.

The contribution of this study revealed new knowledge relating to emergency nurses' feelings of loneliness and frustration in the provision of end‐of‐life care, as well as the importance emergency nurses placed on the presence of loved ones when providing end‐of‐life care. Further research is needed to address how to better support the emergency nursing workforce during a pandemic when providing end‐of‐life care.

## Author Contributions

All authors contributed to the conceptualisation and methodology of the research. A.C., K.C., and J.M. conducted the qualitative data analysis. A.C., J.M., and K.C. prepared the original draft. All authors reviewed and substantially edited the original draft to produce the final manuscript for submission.

## Ethics Statement

Ethics approval was obtained from the Monash University Human Research Ethics Committee (Project Number: 36816).

## Conflicts of Interest

The authors declare no conflicts of interest.

## Peer Review

The peer review history for this article is available at https://www.webofscience.com/api/gateway/wos/peer‐review/10.1111/jan.16749.

## Data Availability

Research data are not shared (due to ethical restrictions).
